# Control and regulation of S‐Adenosylmethionine biosynthesis by the regulatory β subunit and quinolone‐based compounds

**DOI:** 10.1111/febs.14790

**Published:** 2019-03-04

**Authors:** Jiraporn Panmanee, Jack Bradley‐Clarke, Jose M. Mato, Paul M. O'Neill, Svetlana V. Antonyuk, S. Samar Hasnain

**Affiliations:** ^1^ Molecular Biophysics Group Institute of Integrative Biology Faculty of Health and Life Sciences University of Liverpool UK; ^2^ Metabolomics Unit CIC bioGUNE, CIBERehd Parque Tecnologico de Bizkaia Derio Spain; ^3^ Department of Chemistry School of Physical Sciences University of Liverpool UK

**Keywords:** allosteric regulator, colon cancer, hepatocellular carcinoma, methylation, protein–protein interaction

## Abstract

Methylation is an underpinning process of life and provides control for biological processes such as DNA synthesis, cell growth, and apoptosis. Methionine adenosyltransferases (MAT) produce the cellular methyl donor, S‐Adenosylmethionine (SAMe). Dysregulation of SAMe level is a relevant event in many diseases, including cancers such as hepatocellular carcinoma and colon cancer. In addition, mutation of Arg264 in MATα1 causes isolated persistent hypermethioninemia, which is characterized by low activity of the enzyme in liver and high level of plasma methionine. In mammals, MATα1/α2 and MATβV1/V2 are the catalytic and the major form of regulatory subunits, respectively. A gating loop comprising residues 113–131 is located beside the active site of catalytic subunits (MATα1/α2) and provides controlled access to the active site. Here, we provide evidence of how the gating loop facilitates the catalysis and define some of the key elements that control the catalytic efficiency. Mutation of several residues of MATα2 including Gln113, Ser114, and Arg264 lead to partial or total loss of enzymatic activity, demonstrating their critical role in catalysis. The enzymatic activity of the mutated enzymes is restored to varying degrees upon complex formation with MATβV1 or MATβV2, endorsing its role as an allosteric regulator of MATα2 in response to the levels of methionine or SAMe. Finally, the protein–protein interacting surface formed in MATα2:MATβ complexes is explored to demonstrate that several quinolone‐based compounds modulate the activity of MATα2 and its mutants, providing a rational for chemical design/intervention responsive to the level of SAMe in the cellular environment.

**Enzymes:**

Methionine adenosyltransferase (http://www.chem.qmul.ac.uk/iubmb/enzyme/EC2/5/1/6.html).

**Database:**

Structural data are available in the RCSB PDB database under the PDB ID http://www.rcsb.org/pdb/search/structidSearch.do?structureId=6FBN (Q113A), http://www.rcsb.org/pdb/search/structidSearch.do?structureId=6FBP (S114A: P22_1_2_1_), http://www.rcsb.org/pdb/search/structidSearch.do?structureId=6FBO (S114A: I222), http://www.rcsb.org/pdb/search/structidSearch.do?structureId=6FCB (P115G), http://www.rcsb.org/pdb/search/structidSearch.do?structureId=6FCD (R264A), http://www.rcsb.org/pdb/search/structidSearch.do?structureId=6FAJ (*wt*MATα2: apo), http://www.rcsb.org/pdb/search/structidSearch.do?structureId=6G6R (*wt*MATα2: holo)

## Graphical abstract

Methionine adenosyltransferases (MATs) produce the cellular methyl donor S‐adenosyl‐methionine (SAMe) required for methylation, an essential modification of different biomolecules. Mammalian MATs comprise catalytic (MATα1/α2) and regulatory (MATβV1/MATβV2) subunits. The quinolone‐based compound SCR0915 presumably targets the MATβV2/MATα2 dimer binding interface (red boxes), thus behaving as an allosteric regulator that activates MATα2 when Met or SAMe are at low concentration but inhibits the enzyme when Met or SAMe concentrations are high.

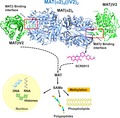

**Abbreviations**
ADO: adenosine
ADP: adenosine diphosphate
AMP‐PNP: adenylyl‐imidodiphosphate
ATP: adenosine triphosphate
LcisAMB: L‐2‐amino‐4‐methoxy‐cis‐but‐3‐enoic acid
MAT: methionine adenosyltransferase
Met: methionine
PPNP: (Diphosphono) aminophosphonic acid
PPPi: tripolyphosphate
SAMe: S‐Adenosylmethionine
SEC: size‐exclusion chromatography
wt: wild‐type

## Introduction

A highly conserved family of Methionine adenosyltranferases (MATs) produces S‐ Adenosylmethionine (SAMe) from methionine (Met) via an ATP‐driven process 1, 2. MAT catalyzes two‐step reactions: (a) SAMe is formed via nucleophilic attack of sulfur atom of methionine to C‐5′ atom of ATP and the tripolyphosphate (PPPi) is generated as an intermediate of ATP cleavage; (b) PPPi is thus hydrolyzed to pyrophosphate (PPi) and orthophosphate (Pi) by tripolyphosphatase activity of MAT. SAMe donates the methyl group to a variety of biomolecules including amino acids, sugars, nucleotide bases of DNA and RNA 3, 4, 5. SAMe is an intracellular regulator of cellular proliferation, differentiation, and death in liver 6, 7, 8 and is involved in gene expression regulation 9, 10. Decrease in SAMe results in abnormal cellular methylation and reduction in antioxidant capacity 11. In mammalian cells, MATα1 and MATα2 are two variants of the catalytic subunit produced from MAT1A and MAT2A gene, respectively. MATα1 functions as homo‐dimer and tetramer^12^, ^13^, while MATα2 forms an hetero‐oligomer with the regulatory subunit MATβ (which has two predominant isoforms differ in lengths, MATβV1 and MATβV2, 334 and 323 amino acids, respectively) in 2 : 1 ratio (MAT(α2)_4_(βV1)_2_ or MAT(α2)_4_(βV2)_2_) 14, 15. MATα2 is ubiquitously expressed in healthy cells, except in the liver (mainly hepatocytes) where MATα1 is primarily expressed, while colon and liver tumor cells ectopically express high amounts of MATα2 16, 17, 18, 19 a switch that promotes the proliferation of cancer cells 20, 21, 22. Although SAMe can be produced by MATα2 alone, the production of SAMe is increased several folds when it complexes with MATβ 14. MATβ interacts with MATα2 through the insertion of the C‐terminal tail of the β subunit into a cavity created at the interface of the MATα2 dimer. Comparison of the MATα2 and MATα2β complex structures revealed that the catalytic site of MATα2 is unaltered upon complex formation thus the increase in SAMe production in MATα2β complex arises from factors beyond the catalytic site through an allosteric regulation 23. The binding of MATβ to MATα2 may propagate from the MATβ‐binding site (Gln190‐Ile198) to regions connecting the active and allosteric site (Ile241‐Tyr271, Gly272‐Thr288), and the region (Ala109‐Gly140) that contains gating loop domain of MATα2 (Gln113‐Gly131). The substrate binding and product release are likely linked to and modulated by MATβ binding to the MATα2 allosteric site offering the use of heterodimeric interface as a target for chemical intervention: either stabilization of defective enzymes or inhibition of the overexpressed enzymes 24.

A gating loop, residue 113–131 in both MATα1 and MATα2, has been identified in the catalytic subunits of all MAT enzymes from prokaryotes to eukaryotes 23, 25, 26, 27, 28, 29, 30. This loop is flexible and presumed to open and close to allow or block access to the active site 26, 31. Recent crystallographic studies of MATα2 and MATα2β complex have shown that the gate is open when active site is empty or occupied by PPNP ((Diphosphono)aminophosphonic acid) and is shut like a lid when the active site is occupied by SAMe or adenosine, demonstrating that the opening of the gate to release product is not driven by hydrolysis of the tripolyphosphate (PPPi) 14, 23.

The majority of amino acid residues that participate in catalysis are conserved in MAT enzymes across a wide range of species, however, the enzyme kinetics of MATs strongly depends on the source of protein 31, 32, 33, 34. The composition of the gating loop varies across species 31. The influence of the length and sequence of the loop on catalytic efficiency has been studied by mutations of *Escherichia coli* MAT gating loop region DRADPLEQ to fragment from rat liver loop sequences (HDLRNEEDV), which resulted in decrease of more than 200‐fold in the maximal rate (*K*_cat_) of SAMe formation, with little effect on the rate of PPPi hydrolysis 35. The active site of MAT is formed at the symmetric dimer interface by mainly hydrophobic interactions except for Arg264 which is one of the few polar residues at the interface. Mutation of Arg264 in MATα1 (Arg264His/Cys) causes isolated persistent hypermethioninemia, which is characterized by low activity of the enzyme in liver and high level of plasma methionine 36. Most genetic mutations are inherited as an autosomal recessive trait 37; however, dominant inheritances of hypermethioninemia have been found in patients with the Arg264 (Arg264His/Cys) and Ala259 (Ala259Val) mutations of MATα1 36, 38, 39. Arg264 facilitates dimerization by providing a positive charge to form salt bridge with Glu57 of the partner subunit and interacts with Gly263 of the partner subunit via K^+^ ion at the central region of dimer interface to form the compact geometry of the active site 40, 41. It has been hypothesized that the abrogated activity of Arg264His/Cys is a consequence of an inability to establish a functional active site 42, 43. Here, we provide direct evidence that mutation does not compromise the dimeric association but results in the deprivation of an interaction at γ‐site of PPPi moiety and the loss of the enzymatic activity in a manner similar to Ser114 mutation, a residue that is part of the gating loop. Ser114 coordinates the adenine ring of the substrate AMP‐PNP (a nonhydrolysable analog of ATP) via a water molecule and provides a rigid helix conformation to the closed gating loop. Surprisingly the mutation of Gln113, the only residue that interacts with Met/SAMe directly, had little effect on the enzymatic activity, as loss of Gln113 does not seem to prevent substrate Met from binding to the active site and the orientation of Met is also preserved. Intriguingly, the phosphatase activity for all of the mutants remains the same as the wild‐type enzyme. High‐resolution crystallographic structures of these mutants together with detailed functional data establish the significance of Ser114 as a sensor of substrate and product. We also show that the loss in activity in Arg264Ala mutant is not due to any compromise in dimer formation but is related to residue's role in the interaction with tripolyphosphate. We show that the enzymatic activity of the mutated enzymes is restored upon complexation with the regulatory protein MATβ and have harnessed this observation to find druggable quinolone‐based compounds that may interact with the interface region utilized in the formation of MATαβ complex and restore the enzymatic activity.

## Results and Discussion

### Enzymatic and phosphatase activity

Using *E. coli* MAT, K_cat_ of SAMe formation has been shown to decrease by more than 200‐fold with mutations in the gating loop without effecting the tripolyphosphate hydrolysis rate 35. Eighteen variants of MATα1 mutation sites have been identified in patients with persistent hypermethioninemia 44, seven of these mutants (Arg249Trp, Ile252Thr, Gly257Arg, Asp258Gly, Ala259Val, Lys289Ash, and Gly381Arg) presented themselves with significantly lower levels of SAMe indicating decreased activity, while their phosphatase activities were unaffected. Taken together, these studies suggested that phosphatase activity is independent on SAMe formation and the gating loop has an important role in SAMe production which does not affect the PPPi hydrolysis rate.

**Figure 1:**
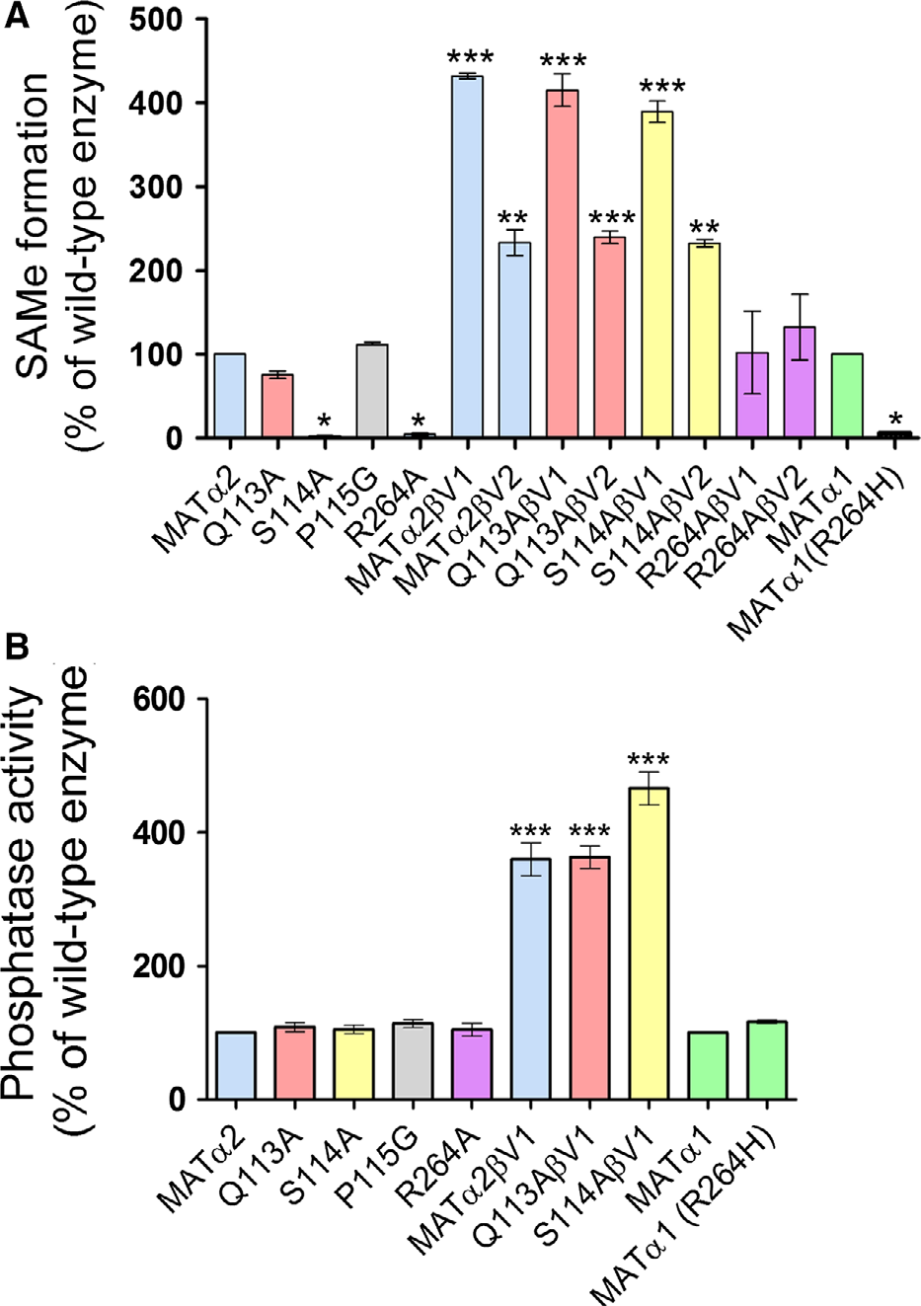
Enzymatic and phosphatase activities of wild‐type and mutant enzymes. (A) SAMe formation of *wt* MATα1 and *wt* MATα2 compared to mutant enzymes (B) Phosphatase activity of wild‐type enzymes and complexes compared to mutant enzymes. (‘*’, ‘**’, and ‘***’ denote statistical significance at *P* < 0.05, *P* < 0.01, and *P* < 0.001 compared with *wt* MATα1/2, respectively). Data are mean ± SEM (*n* = 3).

We explored functional significance of the conserved residues (Gln113, Ser114, and Pro115) among the variable regions of the gating loop using human MATα2 and generating a number of its mutants: Gln113Ala, Ser114Ala, and Pro115Gly mutants. Pro is the only amino acid that connects its side chain to backbone, thus we mutated it to Gly which has no side chain as well, while Gln (Q113) and Ser (S114) have movable side chains and thus more suitable to mutate to Ala to see the functional role of their side chain. We also explored Arg264 residue's role of MATα2 (Arg264Ala) and MATα1 (Arg264His), the mutation of later being mainly affecting to methionine metabolism in hepatocyte of liver and linked to inherited hypermethioninemia. We have shown recently that Gln113 and Ser114 residues interact with Met/SAMe in both the enzyme on its own and when it complexes with MATβ 14, 23. Gln113Ala mutation results in 75.6 ± 4.5% of SAMe production while Ser114Ala mutation results in near abolition of enzymatic activity to only 2.5 ± 0.8% (*p < 0.05*) when compared to *wt*MATα2 (Fig. 1A). In contrast, Pro115Gly mutant has activity similar to the wild‐type enzyme (*wt*MATα2) (Fig. 1A). MATα1 (Arg264His) and MATα2 (Arg264Ala) caused major loss in enzymatic activity with SAMe production reduced to 6.1 ± 2.6% (*p < 0.05*) and 4.5 ± 1.4% (*p < 0.05*), respectively, compared to *wt*MATα1/2 (Fig. 1A).

Remarkably, the SAMe production is recovered in Gln113Ala and Ser114Ala mutants when they complex with either MATβV1 or MATβV2 to the extent that the production of SAMe by Gln113Ala/Ser114Ala MATα2:MATβ complexes was the same as for the *wt*MATα2:MATβ complex confirming the regulatory allosteric role of MATβ (Fig. 1A). The SAMe production by Arg264Ala MATα2 mutant also recovers upon complexation but to the level of *wt*MATα2 on its own. Surprisingly, none of the mutants show any change in their phosphatase activities compared to the *wt*MATα2 (Fig. 1B) providing clear evidence that structural elements that control SAMe synthesis are different from those involved in PPPi hydrolysis. MAT enzyme was suggested to perform ATPase activity as an intrinsic property that functions independently from SAMe synthesis 29, 40. The crystal structure of MATα1 in complex with ADP (Adenosine diphosphate) was derived from the cocrystallization of ATP (Adenosine triphosphate) and methionine analog LcisAMB (L‐2‐amino‐4‐methoxy‐cis‐but‐3‐enoic acid) confirming that the hydrolysis of ATP could take place without SAMe formation 25. Complexation with MATβV1 generally results in higher activity than when MATα2 or its mutants complex with MATβV2. The amount of SAMe produced by Arg264Ala is the same as the wild‐type when it complexes with either of the isoforms of MATβ subunit, thus in this case the effect of MATβ regulatory subunit interaction on functional recovery of Arg264Ala mutant is independent from MATβ lengths. These observations provide clear evidence that interaction with MATβ has a profound effect on the catalytic activity of MAT enzyme where the length of the MATβ protein has a significant impact on wild‐type, Gln113Ala, Ser114Ala (Fig. 1) 45, suggesting that the interaction region may be a candidate for intervention to control and regulate the production of SAMe.

### Crystallographic structures of apo, wild‐type, and mutant human MATα2 enzyme

Seven crystallographic structures are reported here which include apo‐ and ligand‐bound *wt*MATα2, gating loop MATα2 mutants (Gln113Ala, Ser114Ala, and Pro115Gly: Q113A, S114A, and P115G, respectively) and hypermethioninemia‐associated Arg264Ala: R264A mutant. All data collection and refinement statistics are shown in Tables 1 and 2. Structure of S114A mutant has been obtained in two different crystallographic forms (I222 and P22_1_2_1_) while P115G, R264A, and ligand‐bound *wt*MATα2 crystals had I222 symmetry and Q113A, S114A, and apo‐*wt*MATα2 crystallized in space group P22_1_2_1_. Two structures of S114A are identical thus confirming that the detailed structural observations are independent of the crystal form and as such truly represent the molecular behavior of the enzyme providing a rigorous basis for underlying mechanism. The intermediates and products observed in the structures reported here differ (Table 3), although the enzyme, in each case, was incubated with the same substrates Met and AMP‐PNP (adenylyl‐imidodiphosphate, a nonhydrolyzable analog of ATP) prior to crystallization. SAMe is observed in *wt*MATα2, Q113A, and P115G, all of which have similar enzymatic activities (75–110% of *wt*MATα2). SAMe is absent in structures of S114A and R264A mutants which have less than 4% enzymatic activity fully consistent with the inability of these mutants to perform the conversion of methionine into SAMe.

**Table 1:** Crystallographic data collection and refinement statistics for *wt*MATα2 and its mutants

Data collection	Q113A	P115A	*wt*MATα2 (Apo)	*wt*MATα2 (Holo)
Space group	P22_1_2_1_	I222	P22_1_2_1_	I222
Cell dimensions				
a, b, c (Å)	63.63, 103.93, 109.79	66.33, 94.87, 117.37	62.18, 103.23, 108.36	67.63, 94.00, 116.69
α, β, ϒ (Å)	90, 90, 90	90, 90, 90	90, 90, 90	90, 90, 90
Resolution (Å)	103.93–2.70	54.36–2.70	74.74–1.95	73.20–1.35
(last shell) (Å)	(2.83–2.70)	(2.83–2.70)	(2.00–1.95)	1.37–1.35
No. of reflections	20 580	8945	51 446	80 461
*R*_merge_ (%)	14.3 (77.6)	16.2 (56.4)	18.1 (38.7)	8.6 (57.5)
*I*/_Ϭ_ *I*	10.6 (2.1)	5.2 (2.0)	4.7 (2.1)	8.5 (2.1)
Completeness (%)	99.5 (100)	85.0 (89.6)	99.6 (99.8)	98.7 (97.1)
Redundancy	5.6 (5.9)	4.4 (4.3)	3.5 (3.6)	5.0 (4.2)
Wilson B factor (Å^2^)	44.90	51.10	9.94	11.10
Refinement				
*R*_work_/*R*_free**_	17.89/19.02	19.19/23.41	18.96/22.39	13.73/16.19
No. of atoms				
Protein	5711	2977	5711	3089
Ligand	77	50	31	74
Waters	87	3	302	430
Average B‐factors (Å^2^)				
Protein	47.05	49.89	14.66	12.93
Ligand	54.28	54.83	31.43	24.14
Water	33.85	41.8	20.66	28.16
R.m.s. deviations				
Bond lengths (Å)	0.012	0.012	0.013	0.012
Bond angles (^°^)	1.552	1.587	1.666	1.690
PDB Code	http://www.rcsb.org/pdb/search/structidSearch.do?structureId=6FBN	http://www.rcsb.org/pdb/search/structidSearch.do?structureId=6FCB	http://www.rcsb.org/pdb/search/structidSearch.do?structureId=6FAJ	http://www.rcsb.org/pdb/search/structidSearch.do?structureId=6G6R

**Table 2:** Crystallographic data collection and refinement statistics for MATα2 mutants

Mutants	S114A	S114A	R264A
Data collection			
Space group	P22_1_2_1_	I222	I222
Cell dimensions			
a, b, c (Å)	62.09, 102.87, 108.17	66.62, 94.68, 116.55	67.14, 94.23, 116.82
α, β, ϒ (Å)	90, 90, 90	90, 90, 90	90, 90, 90
Resolution (Å)	54.08–1.65	58.28–1.80	58.41–1.70
(last shell) (Å)	(1.68–1.65)	(1.84–1.80)	(1.73–1.70)
No. of reflections	81 704	34 507	40 576
*R*_merge_ (%)	10.8 (56.6)	17.0 (76.6)	12.4 (51.3)
*I*/_Ϭ_ *I*	8.0 (1.9)	5.80 (2.0)	5.9 (1.9)
Completeness (%)	97.2 (98.4)	99.8 (100)	98.7 (99.8)
Redundancy	5.3 (5.3)	5.3 (5.4)	4.3 (4.1)
Wilson B factor (Å^2^)	14.90	17.10	12.40
Refinement			
*R*_work_/*R*_free**_	16.32/19.46	15.10/17.44	16.00/18.42
No. of atoms			
Protein	5902	3005	2963
Ligand	78	79	66
Waters	499	224	284
Average B‐factors (Å^2^)			
Protein	21.72	15.30	15.30
Ligand	27.77	32.78	21.77
Water	27.77	24.81	29.36
R.m.s. deviations			
Bond lengths (Å)	0.015	0.014	0.015
Bond angles (^°^)	1.760	1.670	1.695
PDB Code	http://www.rcsb.org/pdb/search/structidSearch.do?structureId=6FBP	http://www.rcsb.org/pdb/search/structidSearch.do?structureId=6FBO	http://www.rcsb.org/pdb/search/structidSearch.do?structureId=6FCD

**Table 3:** Summary of the enzymatic activities and ligands found in the crystallographic structures for MATα2 mutants and *wt*MATα2

Mutant^a^	Resolution (Å)	Space group	Active site ligands	SAMe synthesis (%)
Q113A	2.70	P22_1_2_1_	PPNP, SAMe	75.6 ± 4.5
S114A	1.65	P22_1_2_1_	PPNP, ADO	2.5 ± 0.7
S114A	1.80	I222	PPNP, ADO	2.5 ± 0.7
P115G	2.70	I222	PPNP, SAMe	111.4 ± 2.8
R264A	1.70	I222	PPi, ADO	4.5 ± 1.4
wtMATα2	1.35	I222	PPNP, SAMe	100.0

a PPPi hydrolysis rate of all mutants are equivalent to wtMATα2 enzyme; polyphosphatase activity is measured in the presence of SAMe.

### Understanding hypermethioninemia and potential therapeutic intervention

**Figure 2:**
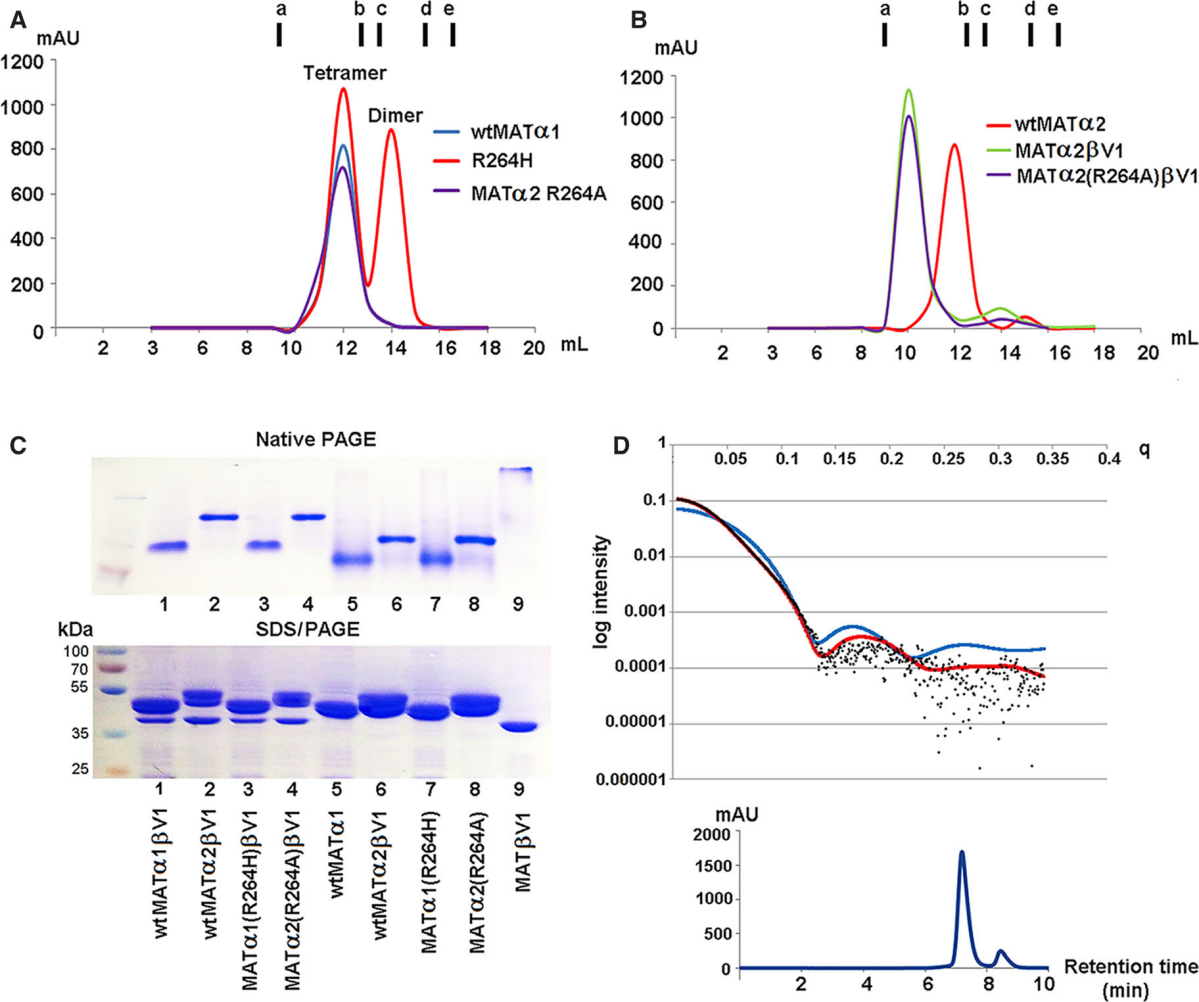
Oligomeric states of MATα2 R264A mutant and MATα1 R264H mutants. Arg264 mutation of MATα1 and MATα2 Gel filtration profiles of (A) *wt* MATα1 R264H and MATα2 R264A mutant; (B) *wt* MATα2–MATβ and MATα2 (R264A)–MATβ complexes compared to *wt* MATα2 alone. Vertical markers (A,B) represent the elution volumes of molecular standard proteins (GE healthcare) including ferritin (a, 440 kDa), aldolase (b, 158 kDa), conalbumin (c. 75 kDa), ovalbumin (d, 43 kDa), and ribonuclease (e, 13.7 kDa) at 9.25, 12.51, 13.32, 14.90, and 16.34 mL, respectively. (C) Native (upper panel) and SDS/PAGE (lower panel) gels of the complexes (MATα1‐MATβV1, MATα2‐MATβV1, MATα1(R264H)‐MATβV1, MATα2(R264A)‐MATβV1) and each individual protein (MATα1, MATα2, MATα1(R264H), MATα2(R264A), MATβV1). For complex formation MATα1 and MATα2 was incubated with both MATβV1 prior to being loaded onto a Superdex 200 10/300. (D) Crystal structure data were used to calculate SAXS profile (red, blue line) and fitted to SAXS experimental data of MATα2 R264A (black dot). X‐ray scattering profile (black dot) is compared with the tetrameric crystal structure (red line, PDB ID: http://www.rcsb.org/pdb/search/structidSearch.do?structureId=5UGH (model); χ = 1.65, C1 = 1.01, C2 = 3.65, Rg = 36.55) and dimeric crystal structure (blue line, PDB ID: http://www.rcsb.org/pdb/search/structidSearch.do?structureId=1QM4 (model); χ = 27.38, C1 = 1.05, C2 = 4.00, Rg = 24.73). The experimental data are a better fit in the tetrameric state with the value of Chi (χ) = 1.65, compared to χ = 27.38 of the dimer for the original crystallographic structure (upper panel). Retention time of MATα2 R264A elution peak is 7.244 min (lower panel).

A number of pathogenic mutations have been identified in human MATα1 which result in the enzyme to metabolize methionine less efficiently. This causes the accumulation of methionine in the blood and in severe cases can develop neurological problems. These sites are found to be conserved in both MATα1 and MATα2. Some missense mutations are found at the dimer interface, for example, Arg249Trp, Ile252Thr, Gly257Arg, Ala259Val, and Arg264Cys/His. In particular, Arg264 forms a salt bridge with Glu57 of the dimeric partner. Its substitution to a cysteine or histidine residue almost completely abolished the enzymatic activity, which was hypothesized to arise from an inability to dimerize 42, 43. In the homologous rat enzyme, replacement of the equivalent Arg265 by His (R265H) resulted in a monomeric species with only 0.4% of the SAMe synthetic activity 43. In contrast, we find that human Arg264His (R264H) MATα1 and Arg264Ala (R264A) MATα2 mutants are multimeric in solution. Two fractions of R264H MATα1 eluted from size‐exclusion chromatography (SEC) correspond to tetramer and dimer, respectively, while *wt*MATα1 and R264A MATα2 eluted primarily as a single tetramer fraction (Fig. 2A). Equivalent mutations (R244L/R244H) carried out in *E. coli* MAT also showed a tetrameric assembly in solution 46. In addition, both R264H MATα1 and R264A MATα2 are capable of forming a hetero‐oligomer with MATβ as shown by analytical SEC for R264A MATα2 in Fig. 2B. The complex formation has been also confirmed by native and SDS/PAGE (Fig. 2C). To establish the oligomeric state of the R264A mutant in solution, small‐angle X‐ray scattering (SAXS) data of purified R264A proteins were obtained at the SEC‐SAXS facility SWING at the SOLEIL synchrotron. The SAXS data (Fig. 2D) clearly showed dominant species to be a tetramer. Although mutants of this residue in both MATα1 and MATα2 form a homo‐dimer in solution, they have no ability to produce SAMe without MATβV1/V2 subunits, which are not coexpressed with MATα1 in the adult liver. The loss of activity caused by point mutation in Arg264 residue can be recovered to *wt*MATα2 level when R264A forms hetero‐oligomer with MATβV1/V2 (Fig. 1A). The observation that the interaction with MATβV1/V2 can modulate and restore function of the pathogenic mutant enzyme activity offers possibility of chemical intervention in finding therapeutic solutions using recently discovered the effect of compounds with triazoloquinoline cores on *wt*MATα2 24.

**Figure 3:**
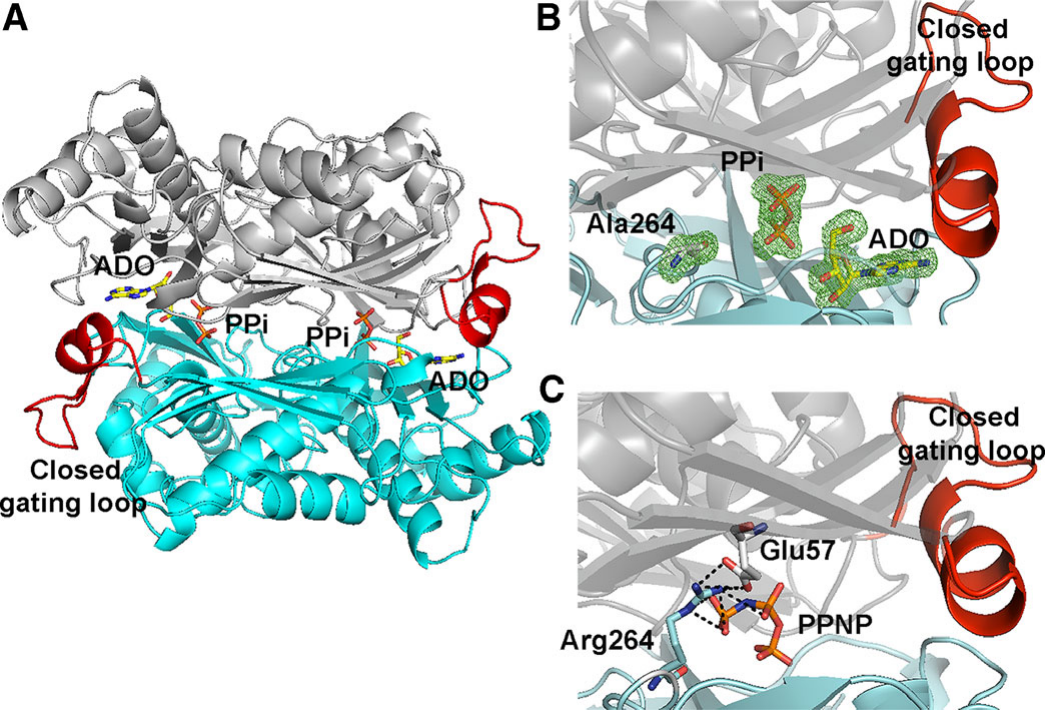
Comparison of R264A MATα2 mutant and wild‐type structure. (A) Cartoon representation of the crystal structure shows fully symmetrical crystallographic dimer (gray and blue) with closed gating loop. (B) A close view of R264A mutant active site shows the omit map contoured around Ala264, PPi and ADO. Fo‐Fc omit maps are contoured at the 3 σ level and colored in green. (C) A close view of dimer interface of Arg264 forming a salt bridge with Glu57 of the dimeric subunit and the interactions of PPNP and Arg264 in *wt* MATα2.

We used the R264A MATα2 mutant as a model to study the catalytic role of Arg264, which was conserved among MATα1 and MATα2. Crystallization of MATα1 has been problematic and attempts to crystallize R264H MATα1 gave only low diffraction quality crystals (the highest resolution we obtained was 3 Å, which is not sufficient to see atomic details). We thus switched to R264A in MATα2, which diffracted at high resolution (1.7Å) with enzyme substrate/product bound and well defined in the active sites. This has allowed us to use the R264A structure as the model to understand the functional role of Arg264 in MAT enzyme. The crystallographic structure of R264A MATα2 revealed the presence of ADO (occupancy, 1.0) and Pyrophosphate (PPi) (occupancy, 1.0) in the active site (Fig. 3A,B). Neither SAMe or Met are observed in this structure. Even though the dimerization is not prevented by Arg264 mutation, the absence of an interaction between NH2 and NE atoms of Arg264 and O2G and O3G atoms of PPNP (Fig. 3C) results in Pγ of PPNP not being observed in the structure, suggesting the importance of Arg264 in stabilizing triphosphate conformation for favorable nucleophilic reaction. Arg264 contributes to the stabilization of the dimer interface through many interactions, especially by forming a salt bridge with Glu57 of the partner subunit (Fig. 3C). Mutation of Arg264 results in a decrease in enzyme stability of both MATα1 and MATα2 as evident by Tm of *wt*MATα1, MATα1 (R264H), *wt*MATα2, and MATα2 (R264A) which were found to be 50.3 ± 0.1, 47.4 ± 0.2, 47.2 ± 0.2, and 42.2 ± 0.2 °C, respectively.

### Inhibition of substrate turnover by a single mutation S114A in the gating loop

**Figure 4:**
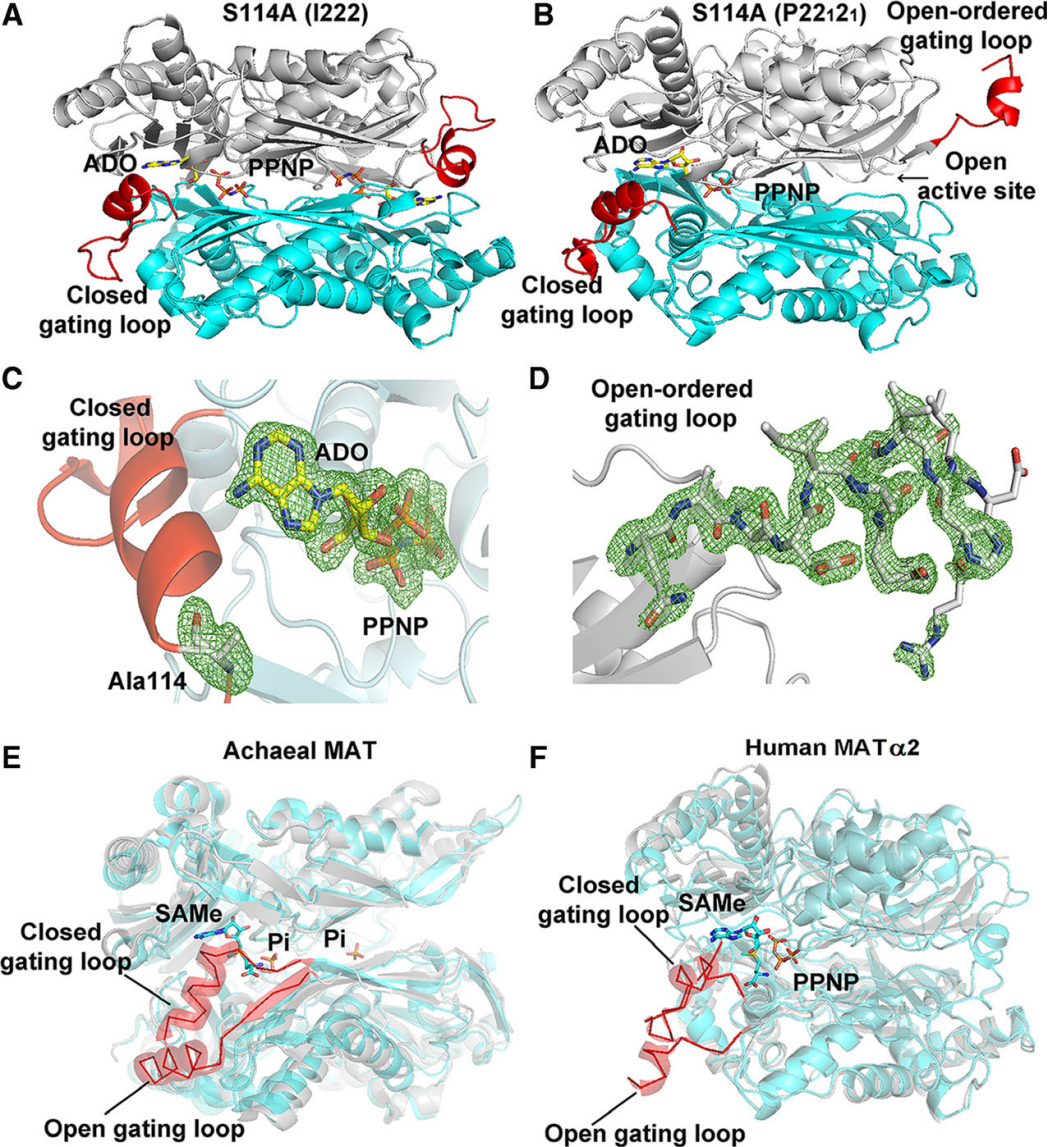
S114A MATα2 mutant structure. (A) I222 crystal form has closed gating loop (red) with PPNP and ADO in the active site. (B) In P22_1_2_1_ crystal form, one monomer shows well‐ordered and closed conformation of the gating loop; ADO and PPNP are bound in the active site, while the other subunit shows a ordered, visible gating loop in the open conformation and no ligand in the active site. (C) Close views of S114A mutation site show the omit map contoured around Ala114 and ligands, ADO, and PPNP. (D) Ordered gating loop (residues 113–125) is in open conformation. Fo‐Fc omit maps are contoured at the 3 σ level and colored in green. (E) Structural alignments of an archaeal MAT in open (PDB: http://www.rcsb.org/pdb/search/structidSearch.do?structureId=4L4Q, gray) and closed gating loop (PDB: http://www.rcsb.org/pdb/search/structidSearch.do?structureId=4L7I, cyan) are shown. Gating loop is shown in red ribbon and all active site ligands (SAMe and Pi) are shown as sticks. (F) Structural alignments of human MATα2 in open (PDB: http://www.rcsb.org/pdb/search/structidSearch.do?structureId=6FAJ, gray) and closed gating loop (PDB: http://www.rcsb.org/pdb/search/structidSearch.do?structureId=5A1I, cyan) are illustrated. Gating loop is shown in red ribbon. SAMe and PPNP are shown as stick.

Two states of the S114A mutant are captured in the space groups I222 and P22_1_2_1_. The crystal structure of S114A in the I222 space group has one monomer in the asymmetric unit. PPNP (1.0) and adenosine (1.0) are clearly visible in the active site (Fig. 4A). The crystal structure of S114A in the P22_1_2_1_ space group has two monomers in the asymmetric unit with different conformations of the gating loop. One monomer has an active site occupied by PPNP (0.5) and adenosine (0.75) and shows well‐ordered and closed gating loop, while the other without any ligand in the active site shows ordered, visible gating loop in the open conformation (Fig. 4B). This is the first time that the gating loop in its open conformation of human MAT enzyme family is clearly visible for the main part of the loop (residues 113–125) (Fig. 4B,D). The same holds true for archaeal MAT that showed structural rearrangement of its gating (flexible) loop (residues 139–156) in apo‐ and holo‐states of the enzyme structure. The open resolved gating loops of *Thermococcus kodakarensis* MAT in apo‐state showed the movement of the gating loop domain (residues 144–155) from its closed conformation (residues 142–142), which is reported in another thermophilic archaeal MAT, *Sulfolobus solfataricus* 27, 30 (Fig. 4E,F). The presence of PPNP and ADO in the S114A structure (Fig. 4C) indicates that the early step of reaction has taken place, but the S114A mutant fails to provide the nucleophilic attack of methionine's sulfur atom against C'5 atom of AMP‐PNP, thus unable to produce SAMe. These observations are consistent with the suggestion that C‐O bond breaking takes place prior to C‐S bond formation at the catalytic transition state of human *wt*MATα2 47. Previously our high‐resolution structure of SAMe+ADO+MET+PPNP *wt*MATα2 (PDB: http://www.rcsb.org/pdb/search/structidSearch.do?structureId=5A1I) had revealed that the Ser114 is the sole residue in the gating loop that interacts with AMP‐PNP via a water molecule. We propose that Ser114 cooperates with water molecule in positioning AMP‐PNP via its adenine ring to the proper moiety, which eventually facilitates enzymatic reaction and SAMe production. Ser114 also interacts with the neighboring loop residues (Asp116, Ile117, and Ala118) and stabilize the helix conformation of the ordered gating loop.

The S114A structure provides information that the gating loop becomes more ordered in the closed conformation as observed by the differences in a helical initiation and termination residue of the closed (Pro115‐His122) and open (Ile117‐Val121) conformation (Fig. 4B): closed gating loop when active site is occupied by ligands which is opened when the active site is empty. In the *wt*MATα2 (PDB: http://www.rcsb.org/pdb/search/structidSearch.do?structureId=5A1I and structure reported here), two active sites are identical, occupied by substrates or products with gating loop in closed conformation, sequestration of reactive intermediates from escaping into solution. In contrast, in both S114A and Q113A mutants (see below), only one active site is occupied by substrate or product suggesting that the gating loop cannot rearrange to the closed conformation properly during the substrate binding step, so the reactive intermediates might be able to leave the enzyme. Two different loop conformations presented in S114A structure indicate that the loop has the ability to adopt different conformations when SAMe formation does not occur.

### Production, control, and regulation of SAMe

Given the dramatic effect of Ser114 mutation on the enzymatic activity of MATα2, mutations of the two neighboring residues Gln113 and Pro115 were undertaken and crystallographic structures determined. As both of these mutants produce a relatively similar amount of SAMe as the wild‐type enzyme, a structural comparison with *wt*MATα2 in its apo‐ and ligand‐bound forms were also undertaken to gain insight into the control and regulation of catalysis.

**Figure 5:**
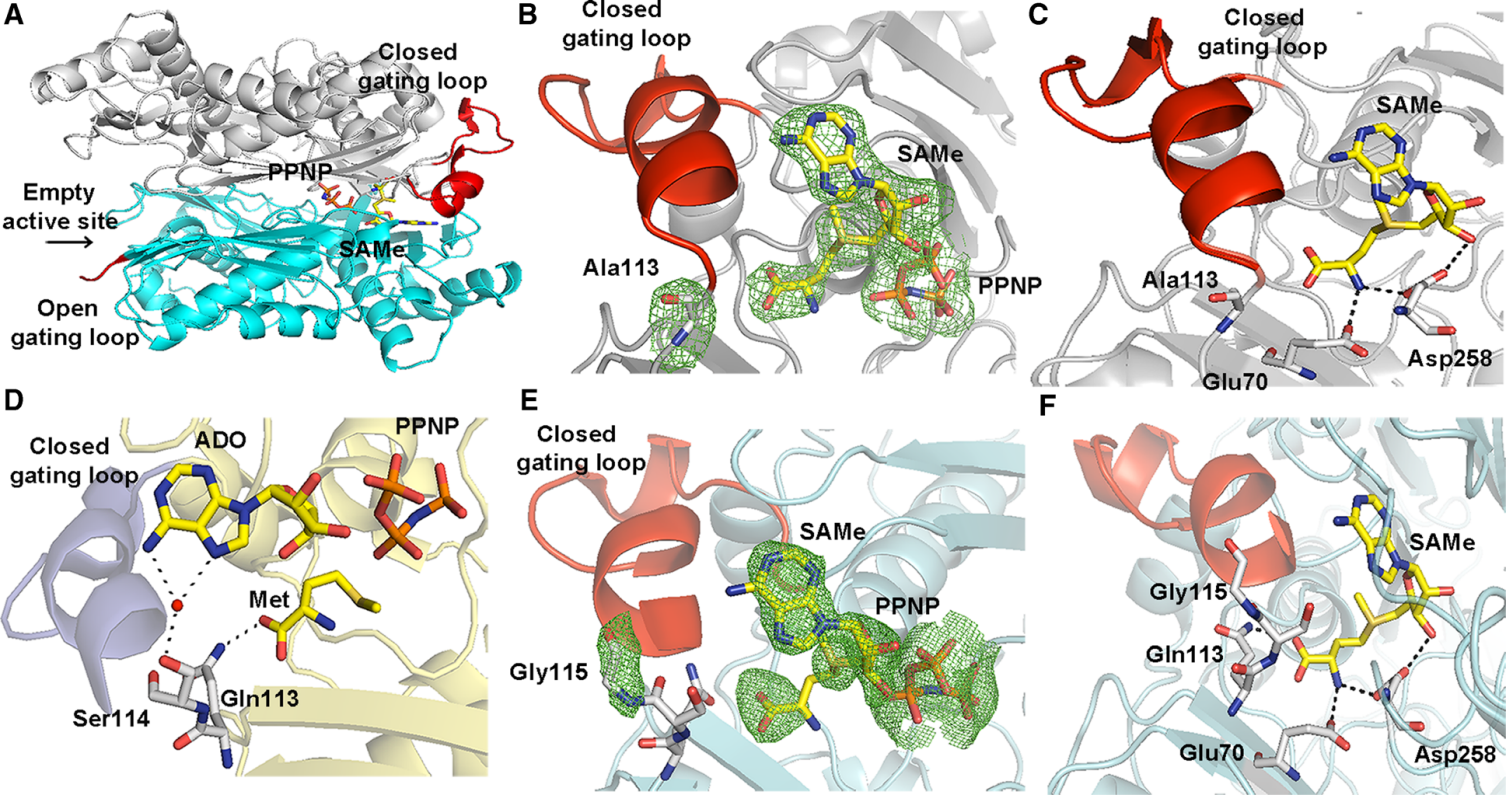
Comparison of Q113A and P115G MATα2 mutant and wild‐type structure. (A) Two different conformations of dimeric subunits of the enzyme are shown. One monomer has a closed gating loop (red) with SAMe and PPNP inside the active site. The other subunit has the disordered, open gating loop (red) and no ligands in the active site. (B) A close view of Q113A mutant shows the omit map contoured around Ala113 and products (PPNP and SAMe) in the active site pocket with ordered and closed gating loops (red). Fo‐Fc omit maps are contoured at the 3 σ level and colored in green. (C) A close view of SAMe in the active site of Q113A mutant. (D) The interactions of Gln113 and Ser114 with Met and ADO in *wt* MATα2. Water is shown as a red sphere. (E) The close view of P115G mutant shows well‐ordered, closed gating loops and the Fo‐Fc omit map is colored in green and contoured at the 3 σ level around Gly115 and products (SAMe, PPNP). (F) A close view of SAMe in the active site of P115G mutant.

The crystal structure of Q113A in the P22_1_2_1_ space group has two MATα2 monomers in the asymmetric unit (Fig. 5A). One of the monomers has closed well‐ordered gating loop with full occupancy of SAMe and PPNP bound in the catalytic site (Fig. 5B). The second monomer has no ligands in the active site, and its gating loop is mostly disordered and does not have any electron density for residues 117–131 (Fig. 5A). In the *wt*MATα2, the N and O atoms of substrate methionine's main chain form hydrogen bonds with the enzyme at OE1 atom of Glu70 and NE2 atom of Gln113 of one subunit, respectively, and its N atom also interacts with OD1 atom of Asp258 belonging to the dimeric partner subunit. Mutation Q113A does not affect the binding of Met and SAMe to the active site. N atom of SAMe forms hydrogen bonds with OE1 atom of Glu70 (2.6 Å) and to OD1 atom of Asp258 (2.5 Å) and O3 atom of SAMe forms hydrogen bond with OD2 atom of Asp258 (3.0 Å) maintaining the position of both substrate and product in the active site (Fig. 5C). The loss of interaction with Met and ADO due to mutation to Ala is likely to be responsible for less efficient SAMe production (75% compared to *wt*MATα2) but the major role of Gln113 is to sense the substrate entry and stabilize the closed gating loop through its interaction with Met, while the entire orientation of the SAMe position is maintained by other residues in the active site pocket as observed from the crystal structure.

The different conformations of the gating loop observed in Q113A and S114A MATα2 mutant structures clearly shows the importance of these two residues in catalysis through regulating the gating loop conformation. These residues sense the presence of substrate Met and ATP in the active site via the interaction of Gln113 with Met and Ser114 with adenine ring of AMP‐PNP, an ATP analog used in the present study (Fig. 5D). These important interactions of the gating loop with the substrates drive the rearrangement of the gating loop to its closed conformation.

The crystal structure of P115G MATα2 displays the closed well‐ordered conformation of the gating loop with full occupancy of SAMe and PPNP in the active site (Fig. 5E). It does not make any contacts with the substrate or the product (Fig. 5F). Despite being a neighboring residue of Ser114, the mutation has no effect on the overall arrangement of the gating loop either. SAMe interacts with Glu70 and Gln113 of one subunit and Asp258 of the partner subunit to maintain itself in the active site pocket. N and O3 atoms of SAMe form a H bond with OD1 and OD2 atoms of Asp258, respectively. The N atom of SAMe also forms a H bond with the OE1 atom of Glu70, while the O atom of SAMe interacts with the NE2 atom of Gln113.

**Figure 6:**
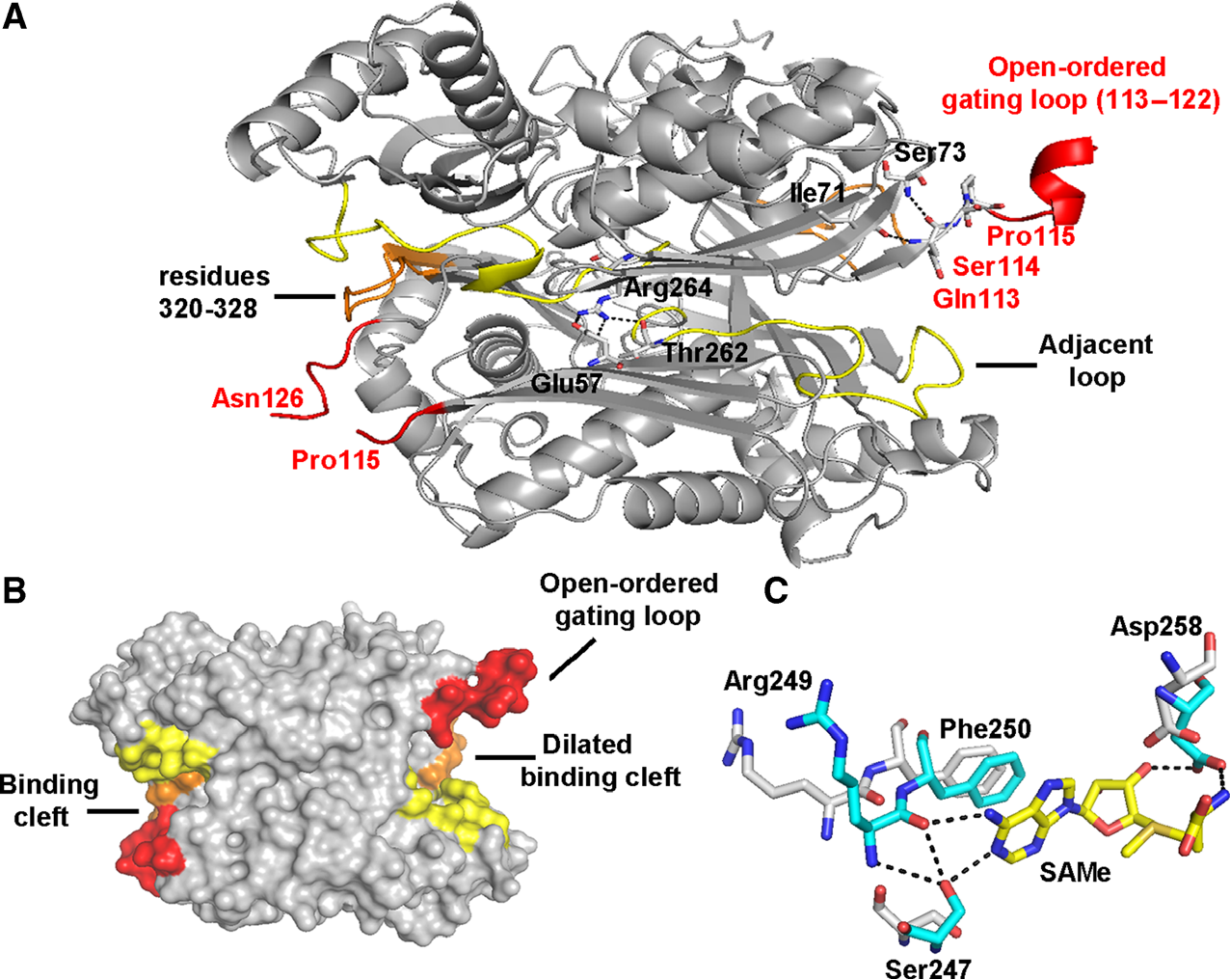
Conformational changes between apo‐ and ligand‐bound *wt* MATα2. (A) Apo‐*wt* MATα2 dimer (gray) shows an ordered and visible gating loop (red) and substrate/product‐binding pocket (yellow and orange). The interactions of the key residues Gln113, Ser114, and Pro115 and R264A are shown in the local surrounding. (B) The surface view of the substrate‐binding clefts of apo‐*wt* MATα2 (gray) is shown with a gating loop (red), adjacent loop (245–264, yellow), and residues 320–328 (orange) (C) Structural comparison between holo‐ (blue stick) and apo‐ (white stick) *wt* MATα2 shows SAMe interactions with Ser247, Arg249, and Asp258 and their positions in the apo‐state.

We have also obtained the structure of apo‐*wt*MATα2 where the gating loops are ordered and visible in the open conformation (Fig. 6A). The structure has a dimer in the asymmetric unit and belongs to P22_1_2_1_ space group similar to S114A mutant. One active site is dilated because of the gating loop, adjacent loop (residues 245–264), and residues 320–328 being oriented in an outward manner to the substrate‐binding cleft, the other subunit features a disordered open gating loop with a less dilated binding cleft (Fig. 6B). Structural comparisons between apo‐*wt*MATα2 and ligand bound (holo) *wt*MATα2, S114A, and R264A have revealed that apart from majority movement in the gating loop region itself, the adjacent loop and residues 320–328 that contribute to the substrate/product‐binding pocket (Fig. 6A,B) of holo‐*wt*MATα2 and R264A line inward to the active site pocket, while these substrate‐binding clefts of S114A are oriented outwardly from the active site similarly found in apo‐*wt*MATα2. These comparisons support the importance of Ser114 interaction with substrates in the rearrangement of the gating loop into its closed conformation and preventing the substrate‐binding pocket residues from moving apart from the active site, which fails in S114A mutant. Most residues that are involved in catalysis including Ser247, Arg249 (Fig. 6A,B) have similar conformation in apo‐ and holo‐*wt*MATα2, except for Phe250 and Asp258. These residues move from their original positions in the apo‐*wt*MATα2 (Fig. 6C). In the holo‐state of *wt*MATα2, the OG atom of Ser247, O atom of Arg249, and OD1 and OD2 of Asp258 form H bonds with N1 (2.81 Å), N6 (2.90 Å), N (2.76 Å), and O3 atom (2.70 Å) of SAMe, respectively, while Phe250 interacts with SAMe through π–π stacking between adenine and aromatic ring of Phe250 (Fig. 6C). The positions of Glu23, His29, Lys181, Lys265, Lys285, Lys289, and Asp291 which are involved in the catalysis are preserved in both apo‐ and holo‐*wt*MATα2. The dilation of the substrate‐binding pocket found in the MAT(α2)_4_(βV2)_2_ complex is identical to the open site found in the current apo‐*wt*MATα2, suggesting that this dilation is not the unique property of the complex.

### Interaction with MATβ and open gating loop in apo‐MATα2

**Figure 7:**
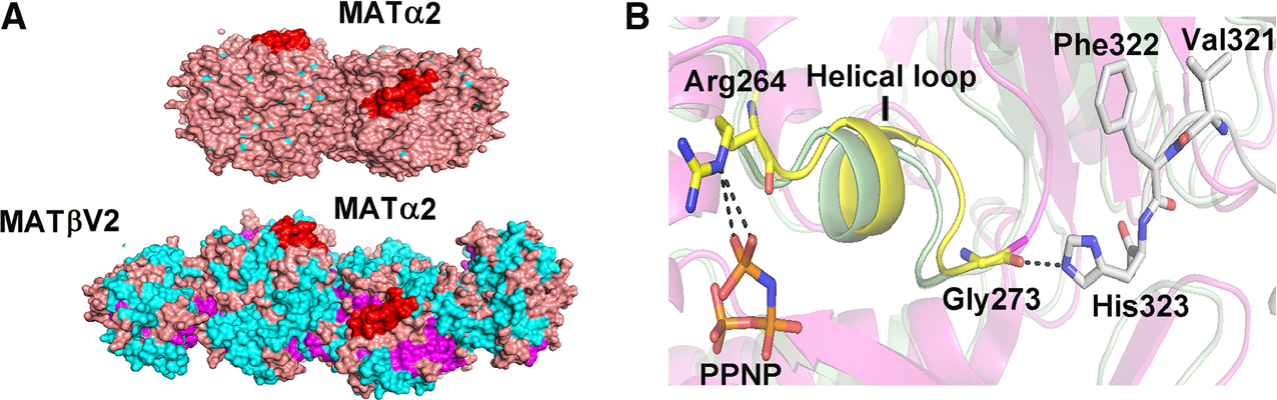
Structural comparison of MATαβ complex and homo‐oligomeric *wt* MATα2. (A) An overview of MATα2 (upper) and MATαβ complex (lower), PDB id: http://www.rcsb.org/pdb/search/structidSearch.do?structureId=4NDN shows enzyme surface with the gating loop colored red, α‐helix, β‐sheet, and connecting loop are shown in cyan, purple, and pink, respectively. (B) A close view of MATα2 in MATαβ complex (pink) and *wt* MATα2 (pale green) alignment of the structures shows the helical loop (yellow), residues 264–273, that recognizes the triple phosphate.

We note that MATβ interacts with the catalytic subunit residues that recognize the PPPi of the ATP in the active site 14 via the helical loop (Fig. 7A,B). NE2 atom of His323 (MATβV2) interacts with O atom of Gly273 (MATα2) of the tripolyphosphate recognition loop (Arg264‐Gly273), where the NE atom of Arg264 (MATα2) interacts with the O1G and O3G atom of PPNP (Fig. 7B). The present study has found that the phosphatase activity of the complexes is significantly higher than that of the catalytic subunits alone (Fig. 1B). Triphosphate is a product intermediate generated from SAMe formation and it has to be hydrolyzed before being released from the active site to allow MAT enzyme to regenerate for the next reaction cycle. The underlying mechanism by which the observed activities of the mutants are recovered by complex formation is in part facilitated by the profound increase in phosphatase activities of the enzyme complexes and is consistent with the protein dynamics study that showed MATα2 ATP‐binding region to undergo allosteric modification upon binding of MATβ 24. Structural comparison of the *wt*MATα2 and MATα2β complex revealed that conformation of the residues in the catalytic site of *wt*MATα2 is unaltered upon complex formation with MATβ; however, several movements of the solvent exposed α‐helices are found in the complex formation. Residues 341–353 are observed prominently being oriented outward when compared to those in apo‐ and holo‐oligomeric of *wt*MATα2. The MATα2β complex structure shows an increase in α‐helix solvent‐exposed area along the MAT(α2)_4_ surface when compared to the homo‐oligomer of the wild‐type (Fig. 7A). Also, the complex stability of both MATα2βV1 (Tm = 55.2 ± 0.1 °C, *P* < 0.001) and MATα2βV2 (Tm = 48.4 ± 0.1 °C) are significantly increased when compared to catalytic subunit alone (Tm = 47.2 ± 0.2 °C, *P* < 0.001). Overall active site conformations for both S114A and R264A mutants are similar to wild‐type protein with 0.2 Å movements of residues 250–286 away from the dimer interface in S114A mutant. Apo‐S114A mutant structure is similar to Apo*‐wt*MATα2, both have a wider open active cleft than in holo‐*wt*MATα2, while in the MATα2β complex this cleft takes an intermediate position (between apo and holo). A less dilated substrate‐binding pocket of MATα2β is a result of the MATβ interaction. The local changes in the complex suggest that MATβ insertion modulates the tightness of the dimer interface (residues 250–286) that comprises substrate‐binding pocket which could result in different degrees of the activity recovery for R264A and S114A mutants.

### Recovery of enzymatic activity through chemical intervention

**Figure 8:**
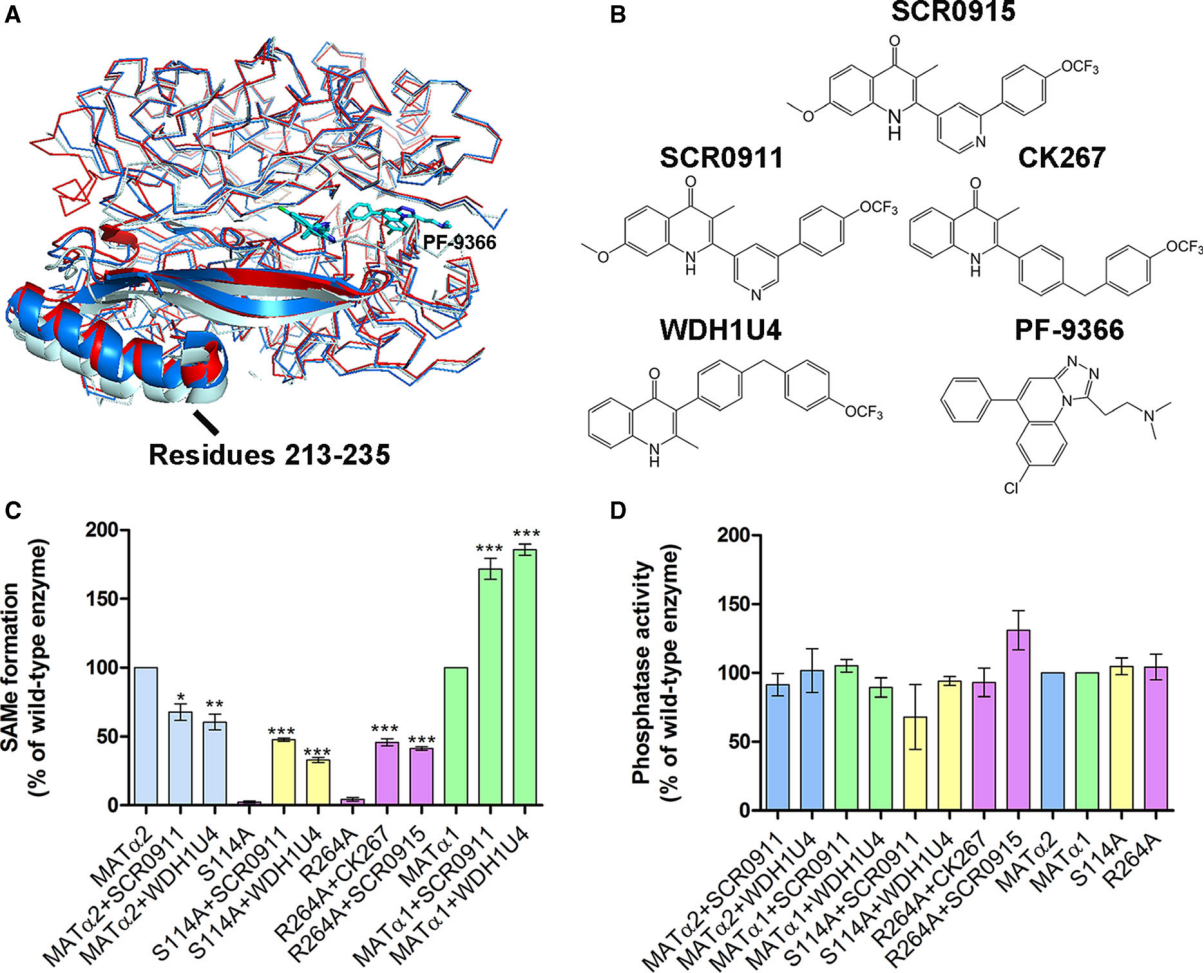
Structural comparison of PF9366‐bound structure to holo‐*wt* MATα2 and MATαβ complex structure and quinolone‐based compound study. (A) PF9366‐bound structure (PDB: http://www.rcsb.org/pdb/search/structidSearch.do?structureId=5UGH, bright blue), holo‐*wt* MATα2 (PDB: http://www.rcsb.org/pdb/search/structidSearch.do?structureId=5A1I, red), and MATαβ complex structure (PDB: http://www.rcsb.org/pdb/search/structidSearch.do?structureId=4NDN, pale blue) are shown. Domain (181–269) with the biggest shift in helical loop (residues 213–235) is shown as cartoon. PF‐9366 is shown as a blue stick. (B) Quinolone‐based compounds used in this study and PF9366 MATα2 inhibitor are shown. (C) Effect of quinolone‐based compounds (10 μm) on SAMe formation of *wt* MAT and mutants. SAMe formation was analyzed by S‐adenosylmethionine ELISA kit (Cell biolabs). All enzymes were preincubated with methionine before adding ATP to initiate the reactions. (D) Phosphatase activity was unaffected by any of the chemical compounds. Data are mean ± SEM (*n* = 3). All reactions of enzyme assays were measured at 750 nm.

The search for chemical compounds that effect the SAMe production of MATα2 enzyme has seen limited success. A recent study 24 showed that 1‐[2‐(dimethylamino) ethyl] quinoline benzodiazepine derivative (PF‐9366) affects the catalysis significantly and competes with MATβ protein by binding in the interaction region that is used by MATβ for complex formation of MATα2β. PF‐9366 binding to Apo‐*wt*MATα2 causes a closure of the interdimer interface similar to holo‐*wt*MATα2 structure with the biggest movement in domain composed by residues 181–269 (Fig. 8A).

We have undertaken a focused screening of quinolone‐based compounds (Fig. 8B) on the enzymatic activities of *wt*MATα1, *wt*MATα2, and MATα2 mutants (S114A and R264A). For the mutant enzymes where the mutation had caused near complete abolition of SAMe production, the activity is restored by these quinolone‐based compounds to varying degrees (Fig. 8C). In fact for *wt*MATα1, the enzymatic activity doubles with a number of compounds while the SAMe production of *wt*MATα2 is marginally reduced by all of these compounds. In contrast, none of these quinolone compounds show any change in the phosphatase activities of *wt*MATα1 and *wt*MATα2 (Fig. 8D), while the binding of MATβ to MATα2 and mutants significantly elevates their phosphatase activities. This selectivity of the quinolone compounds for modulating the SAMe production of the wild‐type and disease‐causing mutants offers a clear opportunity for chemical intervention for therapeutic purposes by altering SAMe levels in the cell.

**Figure 9:**
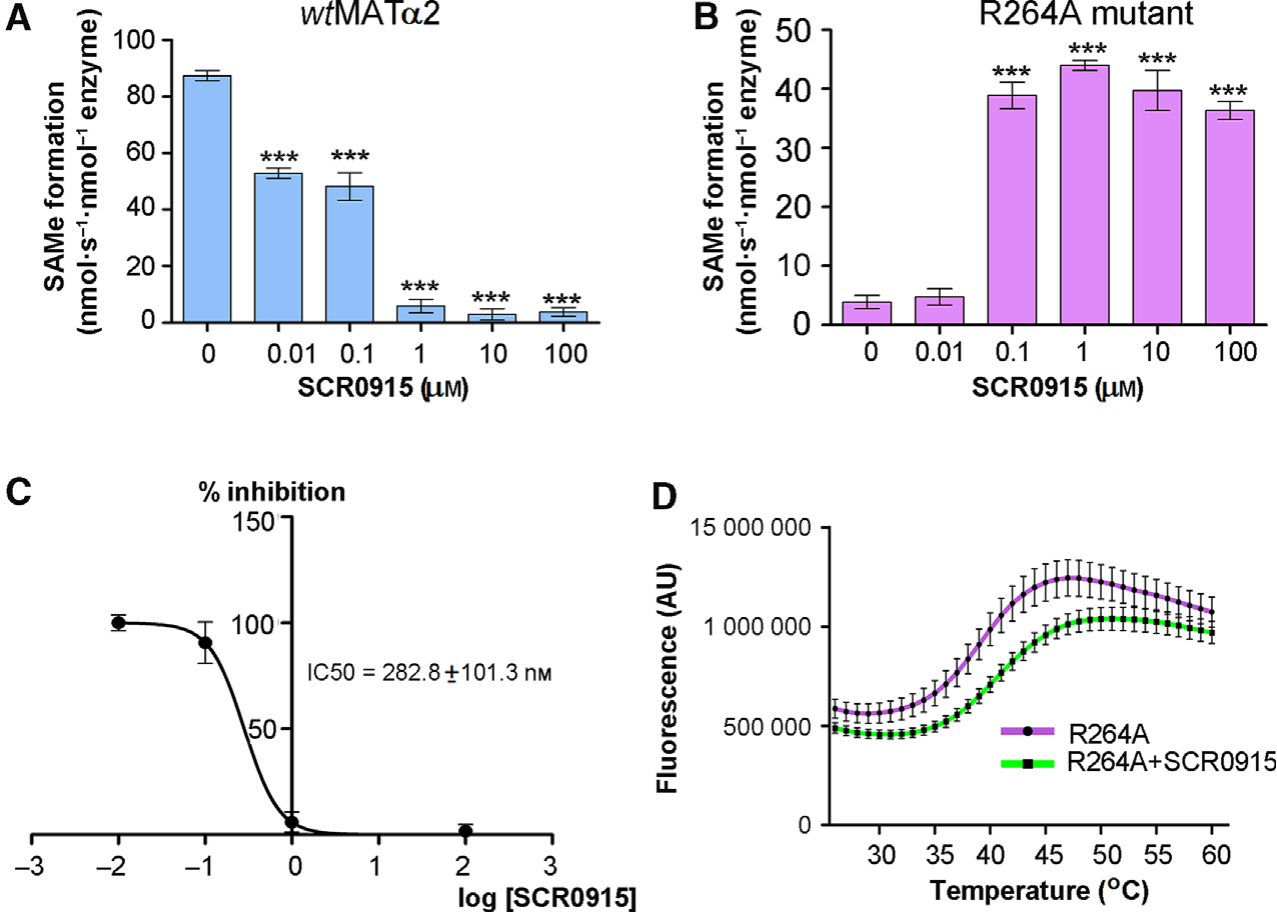
The effect of SCR0915 on *wt* MATα2 and MATα2 R264A activity. (A) SCR0915 inhibits *wt* MATα2 activity in the concentration‐dependent manner (B) SCR0915 recovers SAMe production in R264A mutant. (C) Half‐maximal inhibitory concentration of SCR0915 on *wt* MATα2 is determined (IC_50_ = 282.8 nm). IC50 values were calculated from a log([SCR0911]) versus normalized response curve fit using graphpad prism version 5.00. (D) Thermal shift assay is used to determine R264A stability with and without 1 mm SCR0915 compound. (‘***’ indicates statistical significance at *P* < 0.001 compared with *wt* MATα2 alone). Data are mean ± SEM (*n* = 3).

To gain further insight, we used SCR0915 to study the effect of a quinolone‐based compound on *wt*MATα2 and MATα2 R264A mutant (Fig. 9A–D). In *wt*MATα2, SCR0915 acts as an inhibitor in the concentration‐dependent manner (Fig. 9A). The inhibition curve and IC_50_ of SCR0915 on *wt*MATα2 are determined and shown in Fig. 9C. SCR0915 does not alter the thermal stability of *wt*MATα2. In contrast, SCR0915 could recover R264A activity at 0.1–100 μm (Fig. 9B) and R264A melting temperature increased to 41.48 ± 0.06 °C compared with R264A alone (39.2 ± 0.07 °C), indicating that SCR0915 increases the mutant stability (Fig. 9D). The compound behaves as an allosteric regulator to activate MATα2 when Met or SAMe are at a low concentration, but it inhibits enzyme activity when Met or SAMe concentrations are high. One possible mechanism is that the compound renders MATα2 mutant to be more sensitive to SAMe inhibition, which explains why it decreases the amount of SAMe in *wt*MATα2, and increases SAMe production of MATα2 R264A only to 41.23 ± 1.37% relatively to *wt*MATα2 (Fig. 8C), while MATβ could recover SAMe levels of MATα2 R264A to the same level found in wild‐type (Fig. 1A).

### Molecular modeling of SCR0915‐binding site

**Figure 10:**
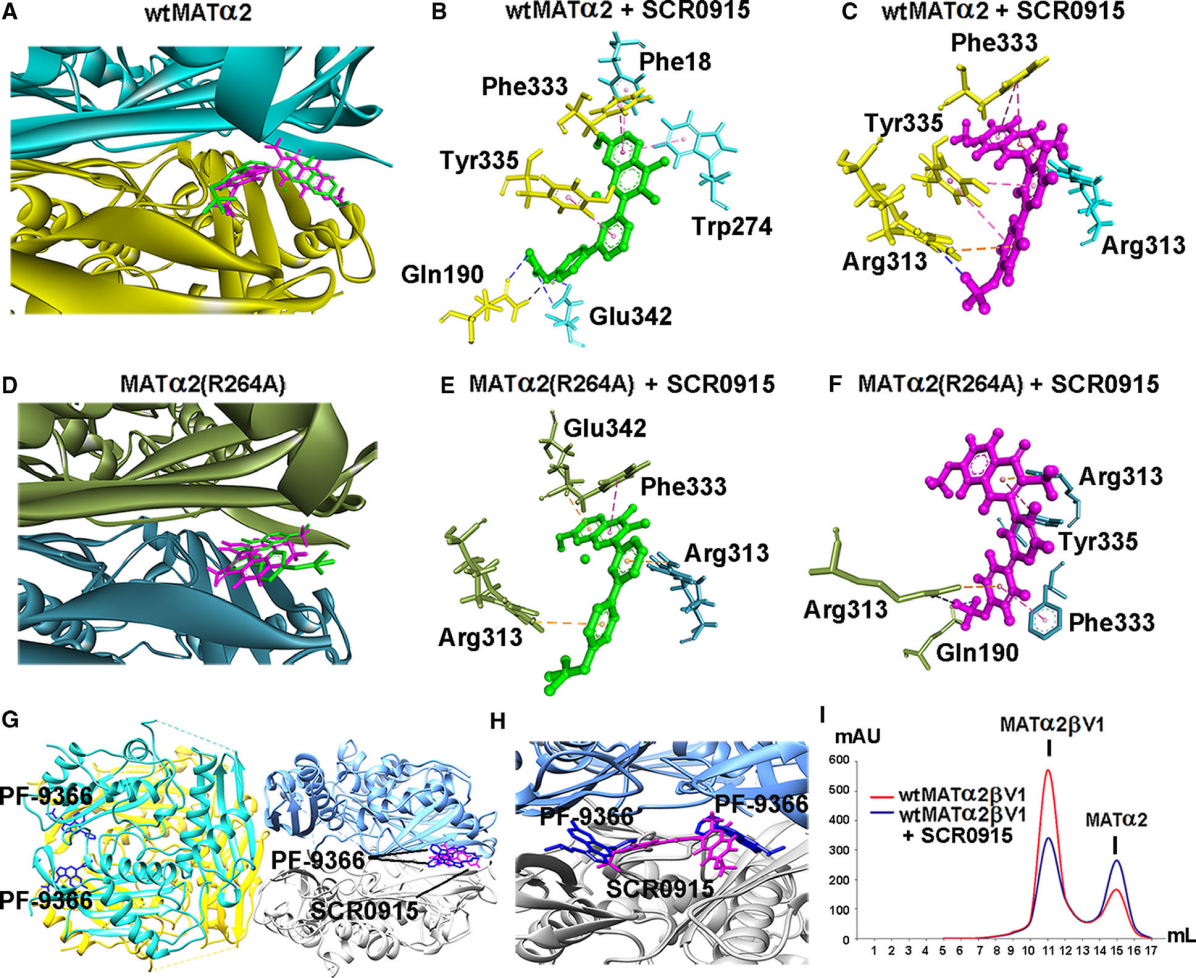
Molecular modeling of ligand‐binding site using Autodock Vina and SwissDock. (A) MATα2 dimeric interface is shown in blue and yellow ribbon. The best pose of SCR0915 binding site is predicted by Autodock Vina and SwissDock. SCR0915 is shown in green stick (Autodock Vina) and magenta stick (SwissDock) (B, C) SCR0915 and wtMATα2 interaction profiles obtained by Autodock Vina and SwissDock are demonstrated, respectively. (D) MATα2 (R264A) dimeric interface is shown in blue and green ribbon. The best pose of SCR0915 binding site is obtained by Autodock Vina and SwissDock. (E, F) SCR0915 and MATα2 (R264A) interaction profiles obtained from Autodock Vina and SwissDock are visualized, respectively. Protein–ligand interactions are shown in dashed line with black (H‐bond), bule (halogen bond), pink (hydrophobic interaction: π–π stacking), and orange color (electrostatic interaction) (G) PF‐9366‐MATα2 structure (PDB, http://www.rcsb.org/pdb/search/structidSearch.do?structureId=5UGH) is shown in tetramer with two molecules of PF‐9366 (blue stick) in each dimer. The modeling of SCR0915‐binding site obtained by SwissDock (magenta stick) overlaps the PF‐9366‐binding site. (H) Close‐up view of the PF‐9366 and SCR0915‐binding site in *wt* MATα2 (PDB, http://www.rcsb.org/pdb/search/structidSearch.do?structureId=5UGH). (I) Gel filtration profile of *wt* MATα2‐MATβV1 complexes is shown. MATα2 is preincubated with 10 μm SCR0915 before forming a complex with MATβV1 (red line). MATα2β complex without SCR0915 intervention is shown (blue line).

The binding of SCR0915 was explored using Autodock Vina and SwissDock using structures of both *wt*MATα2 and MATα2 (R264A). The binding modes are obtained from both local and blind docking and the most favorable energies are evaluated based on Gibbs free energy (∆*G*) in Autodock Vina and FullFitness score in SwissDock. The two docking programs show the best scored positions of SCR0915‐binding site at similar dimeric interface of MATα2 in both *wt*MATα2 and MATα2 (R264A). This dimeric interface‐binding pocket has been reported to interact with MATβ and PF‐9366 compound 14, 24. The best binding position of SCR0915 using *wt*MATα2 structure as a model (PDB, http://www.rcsb.org/pdb/search/structidSearch.do?structureId=5A1I) predicted by Autodock vina (∆*G* = −11.4 kcal·mol^−1^) and SwissDock (FullFitness = −3486.08 kcal·mol^−1^, and ∆*G* = −7.8 kcal·mol^−1^) are shown in Fig 10A–C. The best binding position of SCR0915 using MATα2 (R264A) structure as a model (PDB, http://www.rcsb.org/pdb/search/structidSearch.do?structureId=6FCD) predicted by Autodock vina (∆*G* = −10.2 kcal·mol^−1^) and SwissDock (FullFitness = −3111.79 kcal·mol^−1^, and ∆*G* = −8.1 kcal·mol^−1^) are shown in Fig 10D–F. PF‐9366‐binding structure (PDB, http://www.rcsb.org/pdb/search/structidSearch.do?structureId=5UGH) is shown with SCR0915‐binding site (Fig. 10G). Two molecules of PF‐9366 bound to MATα2 dimer (one molecule to one MATα2 monomer), while one molecule of SCR0915 was predicted to bind to the dimer (Fig. 10G,H). We test whether SCR0915 could inhibit the formation of MATα2‐MATβ complexes by using SEC analysis. MATα2 was preincubated with MATα2 before MATβV1 is introduced into the solution. We find that SCR0915 partially excludes MATβ from binding to MATα2 (Fig. 10I), confirming that SCR0915 interaction sites to MATα2 overlap with the interacting site of MATβ.

## Conclusion

**Figure 11:**
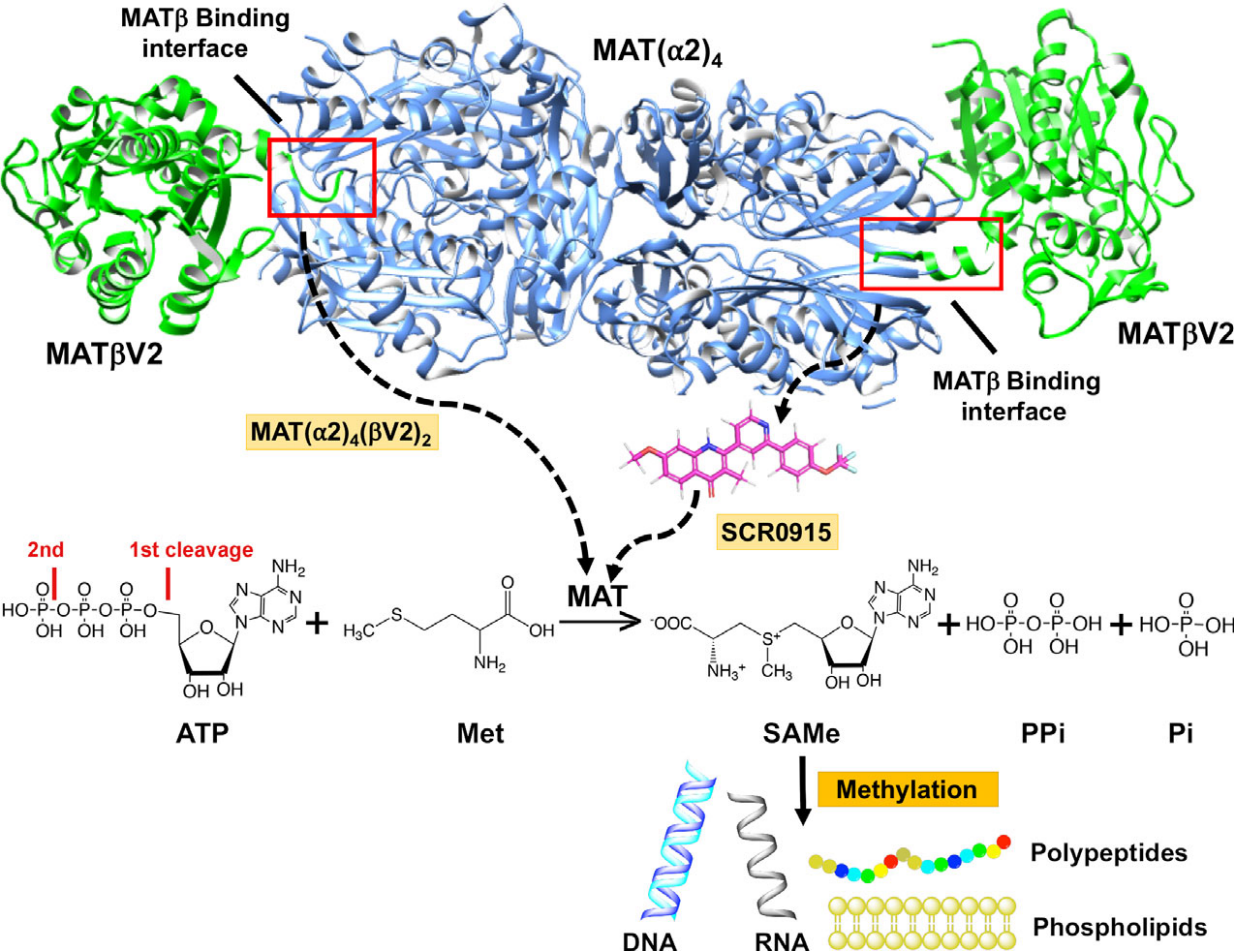
The MATβ and SCR0915 regulate MATα2 activity. The MATβV2‐binding site is located at MATα2 dimeric interface and shown in red box. The compound (SCR0915) might interact with MATα2 at the MATβ‐binding site and behave as an allosteric regulator to activate MATα2 when Met or SAMe are at low concentrations, while inhibiting enzyme activity when Met or SAMe concentrations are high. SAMe levels control methylation process of essential biomolecules (Phospholipids, proteins, DNA, RNA, etc.) of an organism.

The gating loop shows distinct behavior during steps of enzyme catalysis in apo‐, PPNP‐, SAMe‐, Met‐, and ADO‐bound structures providing direct evidence by which the gating loop facilitates the enzyme catalysis. Functional analysis and crystallographic structures in the present study elucidate the nonputative role of the MAT gating loop where the single residue, Ser114, is involved in catalytic reaction by coordinating the adenine ring of the substrate ATP via a water molecule and also providing a rigid helix conformation of the closed gating loop. The observation of the ordered open conformation of the gating loop for the first time provides a clear understanding on how it undergoes structural changes during enzyme catalysis upon substrate binding and product release as observed in Q113A and S114A mutant structures. The interactions of the gating loop with substrates drive the rearrangement to the closed conformation. We also provide the explanation of why Arg264 mutation results in the loss of the enzymatic activity as found in patients with hypermethioninemia. The loss in activity results from the loss of interaction between Arg264 and PPPi moiety at the γ‐site. We also observed that the phosphatase activity and SAMe production involve different parts of the catalytic pocket of the enzyme as phosphatase activity is not affected by the pathogenic mutations where SAMe production is dramatically decreased. A focused screening of quinolone‐based compound that exploits heterodimeric interface as a target for chemical intervention, shows selectivity of the compounds for modulating the SAMe production of the wild‐type and disease‐causing mutant interface (Fig. 11), offering a clear opportunity for therapeutic solutions. The compounds behave as an allosteric regulator to activate MATα2 when Met or SAMe are at low concentrations but inhibits enzyme activity when Met or SAMe concentrations are high.

## Materials and methods

### Site‐directed mutagenesis

Primers (Q113A mat2A F:CTGGTAGCCTTGGAGCAAGCGTCACCAGATATTGCTCAA, Q113A mat2A R: TTGAGCAATATCTGGTGACGCTTGCTCCAAGGCTACCAG, S114A mat2A F: GTAGCCTTGGAGCAACAGGCACCAGATATTGCTCAAGGT, S114A mat2A R: ACCTTGAGCAATATCTGGTGCCTGTTGCTCCAAGGCTAC, P115G mat2A F: GCCTTGGAGCAACAGTCAGGAGATATTGCTCAAGGTGTT, P115G mat2A R: AACACCTTGAGCAATATCTCCTGACTGTTGCTCCAAGGC, R264A mat2A F: GATGCTGGTTTGACTGGAGCGAAAATCATTGTGGACACT, R264A mat2A R: AGTGTCCACAATGATTTTCGCTCCAGTCAAACCAGCATC, R264H mat1A F: GATGCGGGTGTCACTGGCCATAAGATTATTGTGGACACC, R264H mat1A R: GGTGTCCACAATAATCTTATGGCCAGTGACACCCGCATC) for site‐directed mutagenesis were designed by using snapgene software (from GSL Biotech; available at snapgene.com). The desired mutated plasmids were obtained by using CloneAmp HiFi PCR Premix (Clontech, Mountain View, CA, USA). A total of 12.5 μL of CloneAmp HiFi PCR Premix was mixed with 7.5 pmol of each primer, 100 ng of wild‐type DNA plasmid, and adjust volume to 25 μL with autoclaved water. All tubes were centrifuged shortly and the thermal cycler was programmed with the following cycling conditions: 10 min 98 °C; 10 s 98 °C; 15 s 55–60 °C; 45 s 72 °C; 10 min 72 °C. Steps 2–4 were repeated 34 times. DNA templates were removed by adding 1 μL DpnI (NEB, Ipswich, MA, USA) per 25 μL reaction volume, and the PCR products were incubated at 37 °C for 2 h. Then, the plasmids were transformed to Stellar^tm^ (Clontech) competent cells. Transformed cells were plated in selective agar plates and incubated at 37 °C overnight. A single colony was inoculated to 5 mL of selective media, and incubated at 250 rpm, 37 °C overnight. DNA plasmids were purified and sent to Sequencing at GATC biotech, Konstanz, Germany.

### Protein expression and purification of MATα1, MATα2, MATβV1, and MATβV2

MATα1, MATα2 constructs (pNIC28‐Bsa4 plasmid)were kindly provided by The Structural Genomics Consortium (SGC, Oxford, UK), MATβV1 and MATβV2 constructs (pET‐28M‐SUMO3‐GP plasmid) were used in the study. All MAT proteins were produced in *E. coli* BL21 (DE3 strain) and grown in Lysogeny broth (LB) with 50 μg·mL^−1^ of kanamycin for antibiotics selection. Protein expression was induced by adding 1 mm of Isopropyl β‐d‐1‐thiogalactopyranoside (IPTG) at A_600_ = 0.6–0.8. Cells were cultured overnight (18 h) at 20 °C. Cell pellets were resuspended with ice‐cold Lysis Buffer (500 mm NaCl, 5% (v/v) glycerol, 5 mm Imidazole, 10 mm β‐ME). All tubes were placed on ice cell suspension was sonicated by 15 cycles (30 s on/off cycles). Then the sample was centrifuged at 20 000 ***g*** for 40 min. Clear supernatant was collected and load into the His‐trap HP column (GE Healthcare, Chicago, IL, USA) pre‐equilibrated with Lysis Buffer. The column was washed with 10 column volumes of Lysis Buffer and then by five column volumes of Wash buffer (500 mm NaCl, 5% (v/v) glycerol, 30 mm Imidazole and 10 mm β‐ME). Then, proteins were eluted with the Elution buffer (500 mm NaCl, 250 mm Imidazol, 10 mm β‐ME). To cleave the His‐tag from MATα2, eluted proteins were incubated with Tobacco Etch Virus (TEV) protease in 100 (protein):1 (TEV) ratio overnight at 4 °C in Dialysis Buffer (25 mm HEPES pH 7.5, 50 mm NaCl and 10 mm β‐ME). The SUMO tag was cleaved from MATβV1 and MATβV2 by incubating with Sentrin‐specific protease 2 (SENP2) in 1500 (protein):1 (SENP2) ratio. Proteins were left at room temperature for an hour in an orbital shaker and then dialyzed against Dialysis buffer overnight at 4 °C. All proteins were centrifuged at 4000 g for 30 min and loaded onto an anion‐exchange chromatography column (HiTrap Q HP, GE Healthcare) pre‐equilibrated with Buffer A (25 mm HEPES pH 7.5, 25 mm NaCl and 5 mm β‐ME). Proteins were eluted by using salt gradient from 0.05 m to 1 m NaCl (buffer containing Buffer A and NaCl). Selected fractions of proteins were then concentrated by using centrifugal concentrator at 10K cut‐off and loaded onto a HiLoad 16/600 Superdex 200 (MATα1/α2) or HiLoad 16/600 Superdex 75 (MATβV1/V2) gel filtration column (GE healthcare) pre‐equilibrated with 25 mm HEPES pH 7.5. Fractions containing proteins were pooled and stored in 5% glycerol. Proteins were flash frozen by liquid nitrogen and stored at −80 °C.

### Activity assay

The enzyme activity assay was performed by following Murray *et al*. 14. Briefly, all enzymes (MATα2, MAT(α2)_4_(βV1)_2_ and MAT(α2)_4_(βV2)_2_ final concentrations are 50, 25, and 12.5 nm, respectively) were preincubated with 200 μm methionine and reaction buffer (50 mm HEPES pH 7.5, 25 mm MgCl_2_, 25 mm KCl) for 15 min to equilibrate the enzyme and substrate methionine. ATP (1 mm) was added to initiate the reactions and all mixtures were agitated at 1400 rpm (Titramax 100; Heidolph Instruments, Schwabach, Germany), 37 °C for 10 min. The reactions were stopped by adding 50 μL of 100 mm EDTA before measuring SAMe and Pi production. SAMe synthetic activity was measured as the production of SAMe under the saturating concentration of substrates methionine (200 μm) and the constant concentration of ATP (1 mm). Phosphatase activity of MAT enzyme was measured by detecting inorganic phosphate (Pi) under the presence of SAMe. Data were expressed as the means ± Standard Error of Mean (SEM). Significance was assessed using a one‐way analysis of variance (ANOVA), followed by Tukey–Kramer tests using graphpad prism version 5 (GraphPad Software Inc., San Diego, CA, USA). *P*‐values less than 0.05 were considered significant.

### SAMe formation detection

S‐adenosyl‐methionine formation was analyzed by S‐adenosylmethionine enzyme‐linked immunosorbent assay (ELISA) kit (Cell biolabs, San Diego, CA, USA) following the manufacturer's protocol. The measurements were performed triplicates. Data are the average of the independent experiments ± SEM.

### Phosphatase activity

The detection of inorganic phosphate was modified from Baginski *et al*. 48. One hundred and fifty microliter reaction samples were mixed with 150 μL of 2% Ascorbic acid in 10% trichloroacetic acid (A‐TCA). Fifty microliters of water was added in each sample, followed by 75 μL of 2% ammonium molybdate, and 150 μL of 2% (W/V) arsenite–citrate in 10% TCA. All samples were mixed thoroughly (vortex) and incubated at room temperature for 20 min. The samples were measured at 750 nm. Pi quantification was obtained by using a calibration curve obtained from various standard KH_2_PO_4_ solutions covering a Pi concentration range of 0.125–2.0 μL·mL^−1^.

### Quinolone‐based compounds

The synthesis of quinolone‐based compounds has been described by one of the authors in Biagini *et al*. 49 and Charoensutthivarakul *et al*. 50.

### Differential scanning fluorimetry

Differential scanning fluorimetry was performed in a Fast Optical 96‐well reaction plate (Life Technologies, Carlsbad. CA, USA) using Applied Biosystems StepOnePlus™ Real‐Time PCR instrument (Applied Biosystems, Foster City, CA, USA). Each well (20 μL) consists of 10 μL of MATα2 proteins (0.2–1.8 mg·mL^−1^) diluted in 25 mm HEPES pH 7.5 and 10 μL of 10X Sypro Orange (Life Technologies). For quinolone‐based compound study, 10 μL of MATα2 proteins (3.2 mg·mL^−1^) and 1.5 mm of SCR0915 are diluted in dilution buffer (10 mm HEPES pH 7.5, 500 mm NaCl, 5% (v/v) glycerol and 0.5 mm TCEP). Fluorescence intensities were measured from 25 °C to 99 °C with a ramp rate of 1 °C·min^−1^. Data were analyzed using MATLAB^®^ executable TmTool™ following TmTool™ Quick Set‐Up Guide (Life technologies). Data are represented as the average Tm of the independent experiments ± SEM (*n* = 3).

### Crystallization and data collection

All MATα2 mutants were eluted as a single peak by size‐exclusion chromatography. Proteins were concentrated to 5.8 mg·mL^−1^ and pre‐equilibrated with substrates (10 mm Met and 150 μm AMP‐PNP, nonhydrolyzable analog of ATP) in 50 mm HEPES buffer pH 7.5, 10 mm MgCl_2_, 50 mm KCl, and 10 mm DTT before crystallization. Crystal drops containing 1 μL of protein and 1 μL of 100 mm HEPES pH 6.5 and 30% PEG 600 were equilibrated against reservoir solution (100 mm HEPES pH 6.5 and 30% PEG 600). Crystals appeared at 25 °C within 1–2 days. Prior to data collection, crystals were flash frozen in reservoir solution with additional 20% ethylene glycol. Different datasets were collected at the I24, I03, and I04‐1 beamlines at Diamond Light Source (Oxford, England) and the PROXIMA‐1 beamline at SOLEIL synchrotron (Saint‐Aubin, France). Data were integrated by iMosflm 51, 52 and scaled using Aimless 53. The initial model of *wt*MAT2 (PDB: 5A1I) was from crystals with I222 space group. Molecular replacement (molrep software) 54 is needed for mutant structures with P22_1_2_1_ space group, while mutants with I222 space group could be taken straight to restraint refinement using REFMAC 55. Model building and restrained refinement were carried out using coot 56 and REFMAC 55. Crystallographic data collection and refinement statistics and the summary of the enzymatic activities and ligands bound in each structure of MATα2 mutants are shown in Tables 1 and 2.

### Small‐angle X‐ray scattering

Size‐exclusion chromatography–small‐angle X‐ray scattering (SEC‐SAXS) measurements were performed on the SWING beamline at Synchrotron SOLEIL. An Agilent BioSEC Advance 300Å, 4.6 × 300 mm column equilibrated with 25 mm HEPES pH 7.5 was used for a gel filtration step immediately prior to exposure to X‐rays. Twenty microliters of protein at 10 mg·mL^−1^ was injected through an Agilent 1200 HPLC system at a flow rate of 0.3 mL·min^−1^ and column temperature of 15 °C. Exposures were collected prior to the column void volume, for the purposes of buffer scattering subtraction, and over the course of protein elution. Exposures were interspersed for 1 s with a 0.5‐s dead‐time. Frames were inspected and averaged in Foxtrot and analyzed with Primus. The scattering data obtained by SEC‐SAXS are computed and compared to the dimeric and tetrameric oligomer of the crystal structures using FoXS Server 57. FoXS computes the SAXS profile from the input crystallographic structure and fits it onto the experimental profile.

### Molecular docking

Both local and blind dockings are carried out using Autodock Vina 58 and SwissDock 59, 60, respectively. Many binding modes are generated either in a box (local docking) or in the vicinity of all target cavities (blind docking). SCR0915 molecule in Tripos Mol2 File Format (.mol2) is generated using Marvinsketch tool (https://chemaxon.com/products/marvin). The crystal structure of *wt*MATα2 (PDB: http://www.rcsb.org/pdb/search/structidSearch.do?structureId=5A1I) and MATα2 (R264A) (PDB: http://www.rcsb.org/pdb/search/structidSearch.do?structureId=6FCD) which all ligands are excluded is used as a target protein model.

In Autodock Vina studies, the similar grid parameters (box center: *x* = −2.43 Å, *y* = 11.29 Å and *z* = −29.28 Å, box size: *x* = 29.08 Å, *y* = 67.52 Å, *z* = 59.57 Å) are determined in both *wt*MATα2 and MATα2 (R264A) models. All binding modes are generated in the determined box with a number of all possible clusters and the most favorable energy binding mode is evaluated based on its binding energy (∆*G*_binding_: kcal·mol^−1^).

In the Swissdock study, the target protein model in PDB file format and ligand in Mol2 format are uploaded onto a web browser interface (http://www.swissdock.ch/docking). Many binding modes are generated in the vicinity of all target cavities (blind docking). Once the docking processes are terminated, all possible binding clusters are available to download and visualized by Viewdock tool in UCSF Chimera suite 61. The best binding pose is obtained by Gibbs free energy (∆*G*) and FullFitness scores.

The best pose of SCR0915 is selected and combined with its model. The protein–ligand complex PDB file format is written by using UCSF Chimera tool. The protein–ligand interaction profiles are generated and visualized by DS visualizer (Dassault Systèmes BIOVIA, Discovery Studio Modeling Environment, Release 2017, San Diego: Dassault Systèmes, 2016).

## Conflict of interest

The authors declare no conflict of interest.

## Author contributions

JP and JBC designed and performed the experiments. SSH and SVA supervised the project to JP and JBC. PON provided the quinolone‐based compounds. All authors contributed to writing of the manuscript. SSH conceptualized the study.
